# Why is health care so expensive in the United States?

**DOI:** 10.1120/jacmp.v17i3.6426

**Published:** 2016-05-08

In the summer of 1998, I accepted what likely has turned out to be my final professional position in medical physics. When I arrived, my health insurance cost was about $240.00 per month, covering only my spouse and myself. Today, the cost for approximately the same policy has exploded to over $900.00 per month. None of you would be surprised at this; almost everyone in the United States has seen a similar rise in health‐care insurance expenses. For most of us, this insurance is deducted from our paycheck and we see the amount on our paystub.

Compounding this problem is the fact that, not only is the average US health‐care cost more than twice the average of other developed nations, but it is rising faster than any other developed nation (see http://www.commonwealthfund.org/publications/issue‐briefs/2015/oct/us‐health‐care‐from‐a‐global‐perspective).

It is difficult to think of another industry where there is such a lack of transparency respecting the cost to the consumer, and the quality of the product that is delivered. If we buy a car or TV, there is ample opportunity to assess both the cost and quality of the product. In contrast, a patient never knows the true cost; it is lost in insurance, copays, deductibles, payer‐provider contracts, etc., not to mention the fact that you often get bills from multiple entities for the same procedure. The lack of transparency in quality data is even more concerning. How can we possibly tell if a doctor or hospital is any good? Then add the fact that often it is the insurance company that decides where we go, or at least, for whom they will pay. There are usually limited options available through employers because of the deals that employers make with payers.

This Editorial is not intended to be a comprehensive or rigorously reasoned critique of the US health‐care system but, instead, a synopsis of some personal thoughts and insights. As some of you know, I began a long trek toward a second PhD in 2002, eventually finishing it in 2013. Along the way, I took a number of courses in Health Policy and Health Administration. I studied the health‐care systems of many of the other developed nations. I found that, while there were many differences between the various approaches to covering health‐care costs, they all seemed to arrive at about the same place respecting cost and quality of service. All, that is, except for the United States.

Between 2007 and 2010, I served on the Advisory Panel on Ambulatory Patient Classification Groups (Center for Medicare and Medicaid Services), the so‐called APC Panel. I possibly have been the only medical physicist to serve in this capacity. This Panel advises CMS on the valuation of CPT Codes and their grouping within the APC categories, using certain rules. It seeks to advise CMS whenever there is a controversy about the valuation of codes that might unfairly benefit or penalize a drug or device manufacturer or create a barrier inhibiting Medicare patients from receiving services. I was hoping these experiences would help me understand why health care in the United States was so expensive. Although these experiences were helpful, it took several more years and some independent study to come to some tentative conclusions. I am by no means an expert in this area, so I offer my thoughts as a starting place for you, should you wish to investigate this topic on your own.

My first insight, which happened in 2012, was that the US health‐care system financing seemed to work analogous to a reconfigured VAT tax, a consumption tax found in a number of European nations. If you do not know what this is, I found this definition: “Indirect tax on the domestic consumption of goods and services, except those that are zero‐rated (such as food and essential drugs) or are otherwise exempt (such as exports). It is levied at each stage in the chain of production and distribution from raw materials to the final sale based on the value (price) added at each stage. It is not a cost to the producer or the distribution chain members, and whereas its full brunt is borne by the end consumer, it avoids the double taxation (tax on tax) of a direct sales tax. Introduced by the European Economic Community (now the European Union) in the 1970s” (see http://www.businessdictionary.com/definition/value-added-tax-VAT.html#ixzz44oERNuJw).

So, the VAT tax is based on consumption of manufactured goods and services, but what is it used for? Each nation is free to decide just how to allocate its tax revenues. In many countries that have such a tax, the funds are often used to pay for a portion of a nation's health care, usually for the entire population. This structure constrains health‐care revenues to grow somewhat in proportion to the consumption of goods and services in the economy. The VAT tax can be high in countries that use it — as much as 30% of the purchase price of a manufactured item.

It is true that, in the United States, we do not pay such a tax on goods and services, nor do we pay a tax on each component of health‐care delivery, but consider this. Many components of the delivery system (e.g., drugs, devices, construction, salaries, insurance, patient debt) depend substantially on borrowed money. Along with borrowed money comes the fee to service the debt. These fees on debt add end‐to‐end and the result is higher health‐care costs due to debt service. Instead of the government (as would be the case in a VAT tax), large financial institutions are usually the recipients of these debt service fees and the ones that benefit substantially from the current system. The large financial institutions then use these funds to purchase government bond reserves, provide additional loans often for various types of health‐care system loans (such as highly profitable student loans), and of course provide substantial pay packages to its officers.

As an example, of the 10 wealthiest companies with massive debt in the US, two are drug companies, Pfizer and Amgen (http://americasmarkets.usatoday.com/2015/02/05/10‐rich‐companies‐with‐tons‐of‐debt/).

Additionally, much of US health‐care debt is owed directly by US patients — http://health.usnews.com/health‐news/health‐wellness/articles/2014/12/11/how‐could‐america‐get‐its‐medical‐debt‐problem‐in‐check.

I will leave it to you to investigate the debt carried by device manufacturers, the debt held by insurance companies, and the debt accrued by the intermediate and large health‐care systems. All of the servicing of this debt is ultimately carried by the patient as a consumer of healthcare services.

Another insight and an essential factor to consider is the anti‐trust exemption enjoyed by insurance companies, as specified in the 1945 McCarran‐Ferguson Act. The federal law does several things:

- It allows insurers to share related information that lowers costs of doing business. This includes joint development of insurance forms and the sharing of loss data to help with policy pricing;
- It provides insurers with a narrow and limited exemption from federal antitrust laws as long as the activity is state‐regulated;
- It explicitly empowers states to regulate and tax insurance

(See https://www.ohioinsurance.org/factbook2001/chapter6/chapter_6k.htm).

The R Street Institute offers the following thoughts on McCarran‐Ferguson (http://www.rstreet.org/wp‐content/uploads/2015/11/RSTREET46.pdf):

“While explicit price‐and‐wage controls largely have fallen by the wayside in most industries (outside of natural monopolies like utilities), pure rate regulation remains commonplace in insurance. Some degree of rating and underwriting regulation persists in nearly every one of the 50 states. This is, to a large degree, a relic of an earlier time, when nearly all insurance rates and forms were established collectively by industry‐owned rate bureaus, as individual insurers generally were too small to make credible actuarial projections.

“McCarran‐Ferguson charged states with reviewing the rates submitted by these bureaus because of concerns of anticompetitive collusion. With the notable exception of North Carolina, rate bureaus no longer play a central role in most personal lines markets, and many larger insurers now establish rates using their own proprietary formulas, rather than relying on rate bureau recommendations.”

Here are some thoughts from the Alliance for Responsible Legal Funding (http://arclegal‐funding.org/the‐big‐insurance‐equation‐for‐hypocrisy/):

“… in 2010 the sitting Assistant Attorney General of the Antitrust Division at the Department of Justice, Christine Varney, testified before Congress that the exemption is ‘very expansive’ with regard to anything that falls within the business of insurance. This includes premium pricing and market allocations.

“Varney said that insurance companies are ‘virtually always found immune’ of the ‘most egregiously anticompetitive claims, such as naked agreements fixing price or reducing coverage’ due to their antitrust exemption.

“Similarly, the acting Assistant Attorney General from the Office of the Attorney General for New York, Elinor Hoffmann, asked Congress in 2006 to reexamine McCarran‐Ferguson Act because it ‘precludes federal antitrust enforcement of serious anticompetitive conduct’ in insurance. She referenced her department's investigations into ‘new and pervasive instances of abuse’ in the insurance sector, including bid‐rigging and questionable brokerage fees.”

In fairness, this is a very complex issue, and I am sure you would anticipate the insurance industry has a great deal to say about why tampering with or repealing the McCarran‐Ferguson Act would raise rather than lower health‐care costs (http://healthcarereform.procon.org/view.answers.php?questionID=001890).

The Insurance Information Institute would very much like for you to see what they have to say about this: http://www.iii.org/article/antitrust‐law‐and‐insurance.

So I invite you to come to this topic with critical thinking and skepticism and see what conclusion you reach.

The final thought I have is that in the United States, the pharmaceutical industry has achieved a substantial benefit from the Federal Government. CMS is apparently barred from negotiating Medicare prescription‐drug prices in bulk (http://carleton.ca/sppa/wp‐content/uploads/Mirror‐Mirror‐Medicare‐Part‐D‐Released.pdf).

It certainly does not seem to make any sense that the Veterans Administration and Medicaid can negotiate drug prices in bulk, but Medicare cannot. The advantage to the drug companies is estimated at tens of billions of dollars per year and at the expense of higher health‐care costs ultimately for everyone who pays taxes and hopes someday to receive Medicare benefits.

Yes, we as medical physicists in the US enjoy higher salaries and a certain prestige. Additionally, our health‐care benefits are usually quite generous, especially if associated with our employing institutions. However, the path we are on is unsustainable. The growth in health‐care costs, if not checked, will eventually exceed the ability of the US population to service this debt. (See: www.forbes.com/sites/toddhixon/2012/02/09/the‐u‐s‐does‐not‐have‐a‐debt‐problem‐it‐has‐a‐health‐care‐cost‐problem/#61c1c3947f6f).

There is a certain and inevitable adjustment coming to US health care, and I do not believe our profession will be exempt. I submit we should educate ourselves and develop plans to best position the profession in the stormy seas that will characterize the competition for health‐care dollars in the immediate years ahead.

## ACKNOWLEDGMENTS

I want to thank Timothy Solberg and Per Halvorsen, Associate Editors‐in‐Chief of the *JACMP*, for their valuable and perceptive comments.

## COPYRIGHT

This work is licensed under a Creative Commons Attribution 4.0 International License.

Michael D. Mills, PhD

Editor‐in‐Chief

